# Implementing Immunizing Pharmacy Technicians in a Federal Healthcare Facility

**DOI:** 10.3390/pharmacy7040152

**Published:** 2019-11-11

**Authors:** Kimberly McKeirnan, Gregory Sarchet

**Affiliations:** 1 College of Pharmacy and Pharmaceutical Sciences, Washington State University, Spokane, WA 99223, USA; 2 U.S. Public Health Service, Whiteriver, AZ 85941, USA; Gregory.Sarchet@ihs.gov

**Keywords:** pharmacy technician, immunization training, pediatric vaccination

## Abstract

**Background**: Pharmacy technicians are legally allowed to administer immunizations in specific U.S. states, provided they meet certain criteria, including the completion of an accredited immunization training course. Immunizing pharmacy technicians were incorporated into an Indian Health Services federal facility, Whiteriver Service Unit (WRSU), in 2018. The objective of this research was to gather information about implementing immunizing pharmacy technicians in a federal facility serving a large rural and medically underserved population. **Methods**: WRSU launched a Pharmacy Technician Immunization Program in June 2018 after seven federally employed pharmacy technicians participated in the Washington State University accredited technician immunization training. The types of vaccinations administered, and the ages of patients immunized by pharmacy technicians, were tracked from July 1, 2018 to June 30, 2019. **Results**: Seven immunization-trained pharmacy technicians administered 4394 injections for a total of 4852 vaccinations in one year. Vaccinations were administered to patients ranging in age from 2 months old to 85 years old and included protection against diphtheria, tetanus, polio, hepatitis A and B, H. influenza, human papillomavirus, seasonal influenza, meningococcal, measles, mumps, rubella, varicella, pneumonia, and rotavirus. **Conclusion**: In one year, seven pharmacy technicians administered more than 4800 vaccinations to underserved patients. Pharmacy technicians trained and certified to administer immunizations increase access to vaccination care and have the potential to drastically increase the number of immunizations given and reduce the number of deaths from vaccine-preventable diseases.

## 1. Introduction

Medically underserved populations (MUPs) are identified by the Health Resources and Services Administration as populations with a lack of access to primary care services [1]. The calculation for defining a population as medically underserved includes the ratio of providers to population, the percent of the population living at the federal poverty level, the percentage of the population aged 65 years and over, and the infant mortality rate [2]. The work of Murphy et al reported that more than 43% of the U.S. population reside in medically underserved areas (MUA) and that MUAs are not evenly distributed throughout the U.S. [3]. Members of Indian Tribes are automatically designated as MUPs and health professional shortage area (HPSA) populations [4]. An HPSA designation may mean the local population has poor access to primary medical care, dental care, and/or mental health care, while an MUA/MUP designation is based on health status, demographic factors, and the population-to-provider ratio. Immunization rates in American Indian/Alaska native patients are historically lower than other populations. According to the U.S. Health and Human Services Office of Minority Health, American Indian/Alaska Native adults aged 18 years and over are 10 percent less likely than their non-Hispanic white counterparts to receive the influenza vaccine [5]. 

Indian Health Service (IHS) is an agency within the Department of Health and Human Services working to improve the health of MUPs and is responsible for providing federal health services to American Indians and Alaska Natives [6]. IHS provides a comprehensive health service delivery system for approximately 2.6 million American Indians and Alaska Natives, who belong to 573 federally recognized tribes in 37 states. The mission of IHS is to raise the physical, mental, social, and spiritual health of American Indians and Alaska Natives to the highest level, creating healthy communities and quality health care systems through strong partnerships and culturally responsive practices [6].

Whiteriver Service Unit (WRSU) is an IHS facility located on the Fort Apache Reservation, which spans over 1.6 million acres in Northeastern Arizona [7]. WRSU encompasses two The Joint Commission (TJC) facilities, Whiteriver Indian Hospital and Cibecue Health Center. WRSU serves a patient population over 20,000 people, accounting for 40,000 emergency rooms visits and 120,000 outpatient visits annually. Whiteriver Indian Hospital is a 40-bed facility offering outpatient, urgent care, emergency care, optometry, dental, pharmacy, and outreach services. The hospital is staffed by 22 physicians, five nurse practitioners, five dentists, two optometrists, 33 pharmacists, 15 pharmacy technicians, and over 80 nursing staff members. Cibecue Health Center offers outpatient, urgent care, optometry and dental services and is staffed by two physicians, one pharmacist, one pharmacy technician and five nursing staff members. 

**Figure 1:**
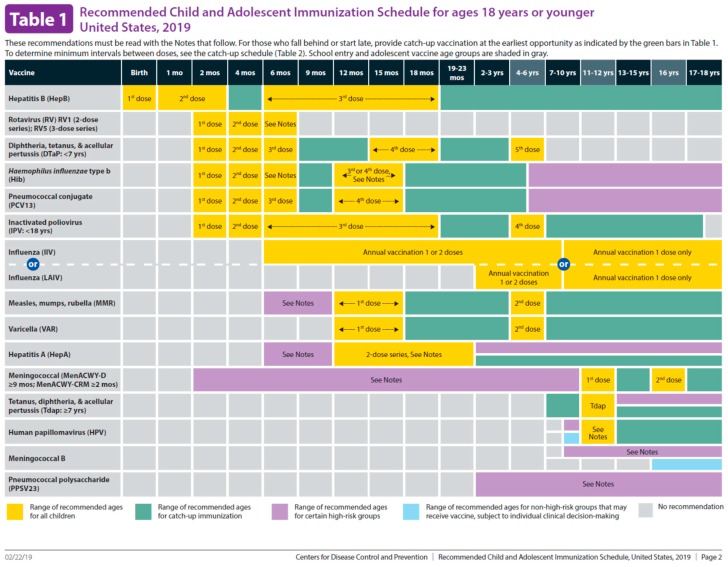
Recommended Child and Adolescent Immunization Schedule for ages 18 years or younger [8].

The Government Performance and Results Act (GPRA) requires federal agencies, including IHS, to measure their performance and effectiveness in improving patient care based on specific standards [8]. GPRA standard measures related to immunizations include ensuring children receive recommended immunizations by three years of age. The recommended child and adolescent immunization schedule is shown in Figure 1 [9]. GPRA measures for childhood immunizations at WRSU have historically been well below the national goal and federal average. WRSU immunization rates also fell below the U.S. vaccination coverage rates for Caucasian children in the age range 19–35 months by almost 30% [10]. As part of an effort to achieve GPRA standards, the pharmacist-in-charge (PIC) at WRSU chose to implement immunization-trained pharmacy technicians as a part of the pharmacy personnel. 

Pharmacy technicians have been shown to be a successful addition to the immunization neighborhood. In 2016, faculty from the Washington State University College of Pharmacy and Pharmaceutical Sciences (WSU) developed the first immunization administration training program specifically for pharmacy technicians [11]. A study by McKeirnan and colleagues in 2018 demonstrated that immunizing pharmacy technicians (IPTs) can be trained to be confident and comfortable administering immunizations after participating in a 4-hour training program [12]. During this study, 25 IPTs administered more than 950 immunizations without incident [12]. In 2019, Bertsch and colleagues stated that pharmacists who supervise IPTs believed the technicians were well trained after participating in the WSU Pharmacy Technician Immunization Training Program, having immunizing technicians increased the number of immunizations administered at the pharmacy, and that training technicians to perform this advanced role had a positive impact on pharmacy morale [13]. Additionally, participating pharmacists believed including an immunization-trained pharmacy technician on the pharmacy team improved workflow and stated they would support immunization training for additional pharmacy technicians [14].

### Objectives

Immunization-trained pharmacy technicians were incorporated into the immunization practices at IHS WRSU and began administering immunizations in June of 2018. The primary objective of this work was to describe the implementation of immunizing pharmacy technicians in a federal facility serving a large rural and medically underserved population. The secondary objective was to collect the number and type of vaccines administered by immunizing pharmacy technicians, as well as the ages of the patients receiving the vaccines.

## 2. Materials and Methods 

In April of 2018, three pharmacy technicians from WRSU traveled to Spokane, Washington to participate in the WSU Pharmacy Technician Immunization Training Program. The program included a 2-hour self-study module, combined with a 4-hour live training session. The WSU training program content is displayed in Table 1. Details regarding administration and completion of the program are described elsewhere [11].

An additional 1-hour pediatric immunization administration module was included, since the WRSU PIC expressed interest in having technicians administer vaccinations to pediatric patients. The 1-hour pediatric modules covered the following skills: patient/parent counseling and comforting; differences between adult and pediatric vaccination doses, injection sites, and supplies; and proper techniques to administer pediatric vaccinations. All three technicians successfully completed the WSU program. In June 2018, four more WSRU pharmacy technicians were also trained using the WSU program. 

In June of 2018, the seven trained immunizing pharmacy technicians were incorporated into pharmacy workflow, including: engagement in pharmacist-supervised, community-wide mass vaccination events; staffing at the walk-in, pharmacy-based immunization clinic; and accompanying pharmacists on home visits to administer vaccines to children who were behind on their vaccinations, including high-risk infants less than one year of age. The types of vaccinations administered, and ages of patients immunized by pharmacy technicians, were tracked from July 1, 2018 to June 30, 2019. 

The 2018–2019 influenza immunization data were also collected and compared to previous immunization records from WRSU. The number of influenza immunizations administered at WRSU was reported annually from October through December, and October through March of the following year. The PIC reported these time periods were chosen to reflect two different periods: the entire influenza immunization season (measured as immunization rates from October through March) and early influenza immunization season (measured as immunization rates from October through December) at WRSU. Influenza immunizations at WRSU typically start in October and end in March and are reported by fiscal year. However, internal facility reports show that, historically, influenza immunizations administered in the early influenza season (October to December) correlate with reduced disease burden seen at the WRSU facilities, so this information was also collected and reported. 

The number of immunizations administered by pharmacy personnel (including pharmacists, pharmacy students, and pharmacy technicians) was also collected and compared with the number of immunizations administered by non-pharmacy personnel. This information was reported quarterly, so trends during influenza and non-influenza immunization seasons were visible. These research methods were found to be exempt from the need for review by the Washington State University Institutional Review Board (IRB application #17571).

## 3. Results

The seven immunizing pharmacy technicians administered 4394 injections and 4852 individual vaccines from July 1, 2018 to June 30, 2019. The number of injections administered and number of specific vaccines administered differ, because several vaccines are available as combination products administered with a single injection. Vaccinations administered included protection against diphtheria, tetanus, polio, hepatitis A and B, H. influenza, human papillomavirus, seasonal influenza, meningitis, measles, mumps, rubella, varicella, pneumonia, and rotavirus. The youngest patient immunized by a technician was two months old and the oldest patient was 85 years old. The number of injections administered by the individual technicians was 749 (TB), 1626 (AN), 38 (Joy T), 171 (MJ), and 1276 (JacT), 55 (EB), 485 (JP), respectively. A description of the number of individual vaccines administered to patients of different ages is included in Table 2.

**Figure 2:**
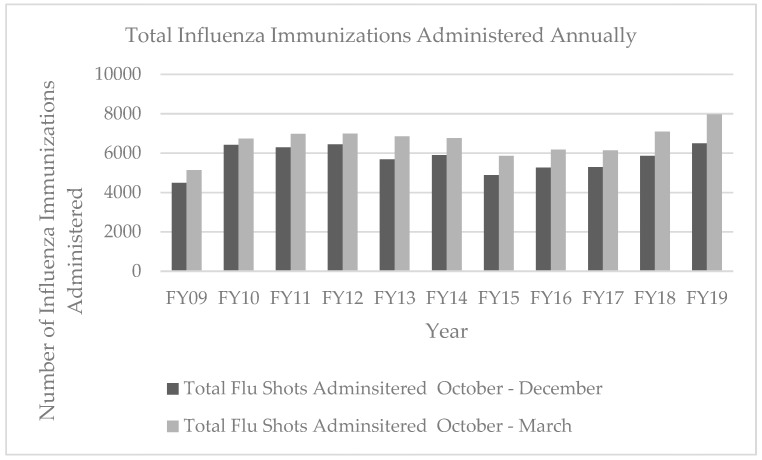
Comparison of the number of total influenza immunization shots administered 2009–2019.

The number of influenza immunizations administered during each fiscal year at WRSU, reported from October to March and October to December, is shown in Figure 2. During fiscal year 2019 (FY2019), WRSU administered 7971 flu shots, 885 more flu shots than were given during any of the previous 10 fiscal years. 

**Figure 3:**
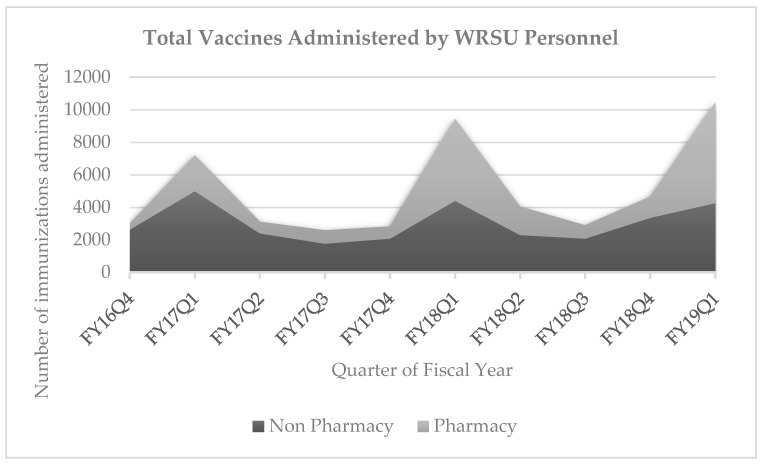
Comparison of annual influenza immunizations administered by pharmacy and non-pharmacy personnel from 2009–2019.

Figure 3 shows the number of immunizations administered by pharmacy personnel, compared with the number of immunizations administered by non-pharmacy personnel. The number of immunizations administered by non-pharmacy personnel remained consistent while the total number of pharmacy-administered vaccinations increased by 1168, when comparing fiscal year 2018 quarter 1 (FY18Q1,) when pharmacy technicians began to administer immunizations, to fiscal year 2019 quarter 1 (FY19Q1), a year later. 

## 4. Discussion

The results of this study demonstrated the effective implementation of immunization-trained pharmacy technicians and the positive impact utilization of pharmacy support personnel can create. Previous research with immunizing pharmacy technicians in Idaho has described similar results, with a large number of immunizations being given by a small number of pharmacy technicians. The supervising pharmacists’ opinions during this project were similar to the results described by Bertsch et al. in 2018: pharmacists support implementation of this advanced technician role and would encourage other technicians to participate in training [13]. During this project, federally employed pharmacists at WRSU commented: “I love having technicians on staff who are immunization certified, it allows me to focus on tasks that require a pharmacist and has decreased our wait times,” and, “Being certified has helped out the workflow positively and greatly. The pharmacists are able to use their time for other complicated patients and administrative matters.”

There has been concern expressed by a few pharmacists, who fear that there is a potential loss of pharmacist jobs and hours, due to the advancement of pharmacy technician practice. At WRSU, the PIC reported utilizing immunization-trained technicians to administer immunizations at mass-vaccination events, in the immunization clinic, and during home visits. As a result of including technicians in the immunizing workforce, the PIC reports that pharmacists were able to see more patients in the patient-centered medical home and chronic disease therapy management clinics, which ultimately allowed both pharmacists and technicians to practice at the peak of their respective licenses. While further research is needed to fully understand the impact of incorporating immunizing pharmacy technicians into the federal pharmacy team, these reports from the PIC indicate that pharmacists are able to provide more clinical services as a result of this initiative. 

Pharmacy technicians play an important role in the pharmacy team. Technicians in all U.S. states can become involved in the immunization process by aiding pharmacists in identifying patients who are eligible for vaccines, assisting with vaccination scheduling, vaccine and supply inventory control, and helping with billing and documentation [15]. In the states of Idaho, Rhode Island, and Utah, and within the federal pharmacy system, technicians are also legally allowed to administer immunizations under the direct supervision of an immunization-trained pharmacist. Ultimately, encouraging pharmacy technicians to participate in an immunization training program provides knowledge enabling the technician to better understand vaccine storage and handling, complete necessary documentation, use regional immunization information systems, utilize immunization schedules, and highlight to patients the importance of vaccinations, regardless of whether or not the technician is physically administering the immunization. WRSU’s leadership supports training technicians in immunization practices, so technicians are actively engaged in many aspects of the immunization process. To this end, the PTEC (Pharmacy Technician Educators Council) and PTCB (Pharmacy Technician Certification Board) have sent letters in support of this initiative in the federal sector. 

Although hundreds of pharmacy technicians have already been trained to administer immunizations using the WSU training program, technicians in this study were the first from Arizona and also the first federally employed pharmacy technicians to become immunization-trained. One challenge to the widespread adoption of utilizing pharmacy technicians to administer immunizations exists in the variation among state pharmacy practice acts, specifically regarding limitations in advanced technician roles. Since states typically define the scope of practice for various healthcare professionals, there is no federal law that addresses this scope for pharmacy technicians. WRSU created a local policy that defined the scope of practice for technicians as it relates to vaccines: if a technician is certified with a state or national pharmacy technician certification and completes the pharmacy vaccine administration training, he or she is able to administer vaccines under the direct supervision of an immunization-certified pharmacist. This example can be extrapolated to other federal sites depending on the local approval structure, interest of pharmacy staff, and governing board approval. 

During the implementation of utilizing technicians as immunizers, questions rose regarding payment for services. Could pharmacies still bill at the same level for immunization services if administration was provided by a pharmacy technician rather than a pharmacist? The resulting conclusion was that the service unit still collected payment for the vaccine administration, since the pharmacist is ultimately ordering the vaccines. This is a similar model used in medical clinics, where vaccinations are administered by a medical assistant or nurse rather than a physician. 

There are limitations to this work. This study describes the efforts of one service unit within the federal pharmacy system, where technician engagement in immunization was supported by the administration. Results would likely differ in federal service units where leadership was unsupportive, or technicians were not given the resources and training needed to implement this innovative service. Additionally, pharmacy technicians in this study were trained to immunize using an accredited immunization program, which had been piloted and refined [11]. Implementation may have faced more difficulty if the technicians involved were not adequately trained to administer immunizations. Finally, technicians in this study immunized many patients in a rural and under-served area. Similar studies conducted in urban areas, or areas that are not under-served, may lead to different results. Additional research in this area is needed. 

There are many opportunities for future research on the topic of immunizing pharmacy technicians in the federal system. It may be beneficial to determine whether demographics, such as technician age, pharmacy work experience, and rank within the federal system, would lead to different results than those seen in this work. Further qualitative work, with an in-depth look at the viewpoints of the immunizing technicians, could also be very telling. Do federal technicians enjoy administering immunizations? Do they find this work fulfilling or is this simply another task that must be done? Learning more about the patient perspective would also be helpful. How did the patients feel about receiving an immunization or watching their child receive an immunization from a pharmacy technician? To truly measure the impact of immunizing technicians, additional data on incidence of vaccine-preventable diseases in this patient population before and after implementation began could also be valuable information. 

Further work is already being done to progress implementation of immunizing technicians within the federal pharmacy system. The USPHS Pharmacy Expanding Vaccine Access (PEVA) Committee is working to provide regional trainings to service units interested in implementing similar programs [16]. The acting PIC at WRSU has already organized several training programs for pharmacy technicians at other regional federal locations, to expand the number of trained technicians within the federal system and encourage adoption of the WRSU model in other pharmacy locations. However, there is still much work to be done to expand the positive results of this study to other federal locations. The impact of incorporating immunizing pharmacy technicians in all IHS facilities or all federal facilities could be quite profound. 

## 5. Conclusions

Between July 1, 2018 to June 30, 2019, seven immunizing pharmacy technicians administered 4852 immunizations with 4394 individual injections to underserved patients. These immunizations included a spectrum of vaccines and patient ages, from routine pediatric immunizations for children less than one year old to zoster and pneumococcal vaccines for older adults. Including pharmacy technicians in the immunization neighborhood has the potential to drastically increase the number of immunizers in the United States, and reduce the number of deaths from vaccine-preventable diseases.

## Figures and Tables

**Table 1:** Content covered during the Washington State University College of Pharmacy and Pharmaceutical Sciences (WSU) technician immunization training program [11].

Topic
Immunization overview
Role of immunizations in public health
Types of vaccines and basic immunology
Common vaccines and vaccination schedules
Tips for identifying patients who have not been immunized
Needle safety and use of sharps container
Routes of administration and finding injection sites
Vaccine handling and storage
Choosing the correct needle and syringe
Drawing up the vaccine for injection
Vaccine administration
Addressing emergency situations
Required documentation and reporting for immunizations
Tips and clinical pearls

**Table 2:** Total vaccines given by pharmacy technicians from July 1, 2018 to June 30, 2019, organized by age and vaccine.

Influenza Vaccines Administered n = 1653	6–23 Months	2–4 Years	5–17 Years	18–49 Years	50–64 Years	Age 65 Years and Older	Total Vaccines Administered
Diphtheria, tetanus, and acellular pertussis (DTaP)	245	92	10	1	0	0	348
Hepatitis A (HepA)	96	39	10	13	11	3	172
Hepatitis B (HepB)	285	11	4	45	26	4	375
*Haemophilus influenzae* type b (Hib)	200	7	0	0	0	0	207
Human Papillomavirus quadrivalent (HPV)	0	0	2	0	0	0	2
Human Papillomavirus 9 (HPV)	0	0	242	94	0	0	336
Seasonal influenza vaccine	80	173	429	600	240	131	1653
Inactivated poliovirus (IPV)	184	92	11	1	0	0	288
Meningococcal (six other strains)	0	0	49	5	0	0	54
Meningococcal B (MenB)	0	0	48	99	0	0	147
Measles, mumps, rubella, varicella (MMRV)	69	84	13	6	6	0	178
Pneumococcal polysaccharide (PPSV23)	0	0	0	94	26	9	129
Pneumococcal conjugate vaccine (PVC13)	245	9	1	3	26	14	298
Rotavirus	104	0	0	0	0	0	104
Tetanus, diphtheria (Td)	0	0	0	1	2	0	3
Tetanus, diphtheria, pertussis (Tdap)	0	0	145	103	42	17	307
Varicella (VAR)	19	1	5	1	0	0	26
Zoster	0	0	0	0	151	74	225
Total vaccines given by age group	1527	508	969	1066	530	252	4852
